# Glycomics@ExPASy: Bridging the Gap*[Fn FN2]

**DOI:** 10.1074/mcp.RA118.000799

**Published:** 2018-08-10

**Authors:** Julien Mariethoz, Davide Alocci, Alessandra Gastaldello, Oliver Horlacher, Elisabeth Gasteiger, Miguel Rojas-Macias, Niclas G. Karlsson, Nicolle H. Packer, Frédérique Lisacek

**Affiliations:** From the ‡Proteome Informatics Group, SIB Swiss Institute of Bioinformatics, Geneva, Switzerland;; §Computer Science Department, University of Geneva, Geneva, Switzerland;; ¶Swiss-Prot Group, SIB Swiss Institute of Bioinformatics, Geneva, Switzerland;; ‖Glyco Inflammatory Group, Department of Medical Biochemistry and Cell Biology, Institute of Biomedicine, Sahlgrenska Academy, University of Gothenburg, Gothenburg, Sweden;; **Institute for Glycomics, Gold Coast Campus, Griffith University, Southport, QLD, Australia;; ‡‡Biomolecular Discovery & Design Research Centre, Macquarie University, North Ryde, NSW, Australia;; §§Section of Biology, University of Geneva, Geneva, Switzerland

**Keywords:** Bioinformatics, Bioinformatics software, Glycoproteomics, Glycosylation, Glycomics

## Abstract

Glycomics@ExPASy (https://www.expasy.org/glycomics) is the glycomics tab of ExPASy, the server of SIB Swiss Institute of Bioinformatics. It was created in 2016 to centralize web-based glycoinformatics resources developed within an international network of glycoscientists. The hosted collection currently includes mainly databases and tools created and maintained at SIB but also links to a range of reference resources popular in the glycomics community. The philosophy of our toolbox is that it should be {glycoscientist AND protein scientist}–friendly with the aim of (1) popularizing the use of bioinformatics in glycobiology and (2) emphasizing the relationship between glycobiology and protein-oriented bioinformatics resources. The scarcity of data bridging these two disciplines led us to design tools as interactive as possible based on database connectivity to facilitate data exploration and support hypothesis building. Glycomics@ExPASy was designed, and is developed, with a long-term vision in close collaboration with glycoscientists to meet as closely as possible the growing needs of the community for glycoinformatics.

Glycoscience is gaining recognition as an important component of life science, as emphasized in two recently published roadmaps issued by the American National Research Council in 2012 (1) and by the European Science Foundation (ESF) GlycoForum (http://ibcarb.com/wp-content/uploads/A-roadmap-for-Glycoscience-in-Europe.pdf) in 2015. Both references point to the same need for organizing access to glycan-related data that is absent in current bioinformatics resources.

Glycosylation is the most common protein post-translational modification yet its role is far from being understood. Glycans, proteins to which they are attached (glycoproteins) and proteins to which they bind (lectins or carbohydrate-binding proteins) are the main molecular actors in this overall cell surface picture as well as the enzymes that are needed to synthesize or trim the attached glycans. The first challenge in collecting this data is the wide range of experimental techniques used to analyze glycans and to elucidate their biological roles. Mass Spectrometry (2) and Nuclear Magnetic Resonance (3) methods are commonly used to solve glycan structures released from proteins. High or Ultra-High Performance Liquid Chromatography (4), and Capillary Gel Electrophoresis with Laser-Induced Fluorescence (5) experiments are used for high-throughput separation of released and labeled glycan structures for determination of their expression. Molecular Dynamics (6), Isothermal Titration Calorimetry or Surface Plasmon Resonance (7) are key techniques to track glycan-protein interactions, as well as glycan and protein/lectin arrays (8, 9). However, all these approaches are used in rather low throughput to date. Mass spectrometric proteomics approaches are recently being used to determine glycan compositions at specific sites on proteins (glycopeptide identification as reviewed in (10)) at higher throughput but at present there is a modest amount of data in glycomics in contrast to most other -omics.

After assessing the spread of the data, the next major obstacle lies in linking together this data that is usually acquired independently. As it is, most glycan structures have been solved after being cleaved off their natural support whereas protein glycosylation sites are identified after fully removing or partly trimming down the attached glycans or with only the monosaccharide composition determined. As a result, key information on the glycoconjugate is lost. Furthermore, results relative to glycan-binding are in most cases obtained with free glycans. The correlation between glycan structures and glycoproteins can be sometimes extracted manually by literature searches but this data is limited and spread over many publications and is recorded in a range of different formats and representations. The limited usage of existing standards for encoding and representing glycans makes the extraction and collation of information labor intensive and time consuming (11). In the end, glycan, glycoprotein and glycan-binding data has accumulated but at different paces and with poor interrelatedness, when obviously all these parameters need to be connected for understanding of the functional role of glycoproteins.

These requirements highlight the need for piecing the disparate information together and building corresponding analytical tools to facilitate data acquisition and interpretation (12). The recent rise of glycoproteomics analytical methods (10) and the constant development of higher throughput and quantitative glycomics (13) is bringing to the fore the need for dependable glycoinformatics resources. A range of tools is now available for processing glycan structure analytical data mostly from mass spectrometry (14). Spectral annotation is likely to improve when comprehensive structural databases become universally available. This evolution is being influenced by several recent moves toward facilitating information sharing. First, glycan structural data collection can now be given unique identifiers that are supported by the wider community (15). Second, an agreement on the simplified representation of carbohydrates with the Symbol Nomenclature for Glycans (SNFG) has been reached (16). Third, guidelines for recording experimental metadata are being defined through the MIRAGE initiative (17, 18). This momentum needs to persist to bring glycoinformatics to a more mature stage compatible with larger scale glycomics and glycoproteomics studies. We recently reviewed the current status of the field (19).

The range of existing, as well as future, glycoinformatics resources warrants dedicated portals as initiated many years ago by GLYCOSCIENCES.de (20) and by the Consortium for Functional Glycomics (CFG)^1^. Each proposed specialized toolboxes. Although the former gathered databases and software that were strongly anchored in the chemistry of glycans and glycoproteins, the latter offered comprehensive glycan-binding data. Later, RINGS (21) was launched; it hosts a series of tools based on machine learning and tree mining to classify and align glycan structures as well as utilities for translating glycans structures between different encoding formats. However, in all these initiatives, many of the corresponding web interfaces are rather cryptic outside the glycobiology community. Furthermore, the interruption of financial support for GLYCOSCIENCES.de and reaching the natural end of the CFG project has resulted in freezing further development and creating acute maintenance problems for the corresponding developed portals.

In this context, the important focus of our bioinformatics and glycoscience collaborative initiative was to guarantee on-line resource longevity and try to circumvent issues arising from short-term or restricted funding. We therefore selected a well-established bioinformatics portal, ExPASy (22) that has been hosting the leading proteomics Swiss-Prot knowledgebase, then UniProt, for over twenty years. We have gradually populated ExPASy with developed glyco-related web-based resources and a glycomics tab was created where this new collection, that we have called *Glycomics@ExPASy*, is now growing. We have recently broadened our range by including a selection of reference web-based resources in glycoscience that have been developed elsewhere, thereby increasing the coverage of the many aspects of glycobiology and the usefulness of the portal. This also matches the spirit of ExPASy, which has traditionally catalogued a mix of in-house and external resources in other fields, such as proteomics.

We initially appraised the requirements of an efficient glycoinformatics toolbox to support research in glycomics and glycoproteomics and identified some of the gaps between glycomics and other -omics. We then set ourselves the goal of filling those gaps. Early tasks involved defining and/or selecting data formats and ontologies, structuring data in new databases (23, 24) and implementing new tools (25–27). The philosophy of our initiative is to be {glycobiologist AND protein scientist}–friendly with the aim of facilitating (1) the use of bioinformatics in glycoscience and (2) the relation between glycobiology and protein-oriented bioinformatics resources. The scarcity of this bridging data led us to design tools as interactively as possible based on implementing database connectivity to facilitate data exploration and support hypothesis building.

Many glycomics and glycoproteomics experiments tend to generate results in the form of lists of results; such as lists of glycan compositions or glycan structures, lists of glyco-epitopes or lists of glycoproteins. The Glycomics@ExPASy toolbox is organized to move away from displaying lists and to provide web interfaces highlighting relationships between the molecular entities involved. For example, shared substructures as glycoepitopes (*e.g.* Lewis a as part of Lewis b) are not apparent in a list whereas they can be highlighted if shown as the products of an enzymatic pathway and as components of a bigger glycan structure. Our technical options aim at implementing modular, interoperable and reusable applications to rationalize and speed up development. In addition, the coverage of our resources is designed to reflect the situation at the cell surface where glyconjugates and glycan-binding proteins functionally interact. Glycomics@ExPASy has been listed as a reference on the NCBI glycan page that was associated with the recent third edition of *Essentials of Glycobiology*, as highlighted in (28) but only described as a URL. The present article details for the first time the growing content of the present interactive tool collection destined and designed to expand as glycoinformatics grows. The glycoinformatics resource described here emphasizes the potential of database and tool combination for integrating data and building new hypotheses.

## MATERIALS AND METHODS

**Fig. 1.**
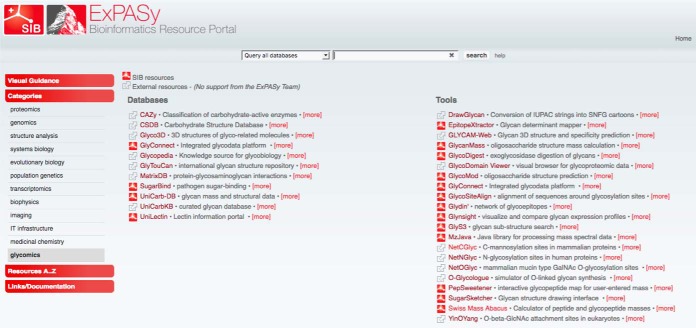
**The glycomics tab of the ExPASy website.** This figure captures the Glycomics tab of ExPASy website. Glycomics resources are divided in two sections: Databases on the left and Tools on the right. In addition, resources developed by SIB are identified with the SIB logo whereas a gray icon precedes external tools. This screenshot reflects the content as of July 2018. The range of databases and tools is destined to expand and this image is likely to differ in the years to come.

As in any other section/tab of ExPASy, *e.g.* proteomics, two lists describe the collection, one for databases and one for software tools as shown in the screenshot of Fig. 1. The purpose of each item of the list is briefly summarized after its name. Our current glycomics and glycoproteomics selection is exclusively composed of databases and web-interfaced tools to the exception of MzJava, a software package used in several of our applications. It was designed for a broad usage in proteomics and glycomics spectral data management (29). MzJava was in fact primarily listed in the proteomics tab of ExPASy where it was popularized. Its occurrence in Glycomics@ExPASy simply highlights the potential for applications in either glycomics or proteomics.

We now describe the principles guiding the development of Glycomics@ExPASy, as well as the technical choices we made for in-house resources.

### 

#### 

##### Formats and Standards

We strive to use the same nomenclature, format and reference sources to encode and describe glyco-related information in our resources as detailed below.

##### Structure Encoding

As explained in (11), several naming schemes have been proposed to describe and represent each monosaccharide. The diversity of encoding of monosaccharides is in most cases summarized in MonosaccharideDB (20). Assuming monosaccharides are clearly defined, the next step is to describe and represent the tree-like structure of glycans. Several attempts have been made to encode structures in a linear format that is computationally advantageous. Such variety of glycan encoding formats has prompted the need for translators (11). To minimize the encoding and decoding phases in Glycomics@ExPASy tools, we chose GlycoCT (30) as our global glycan encoding format. GlycoCT is widely used in the most recent databases and software. All the web interfaces developed for SIB tools, accept GlycoCT as input for glycan structures. All SIB databases (UniCarb-DB, GlyConnect and SugarBindDB) store glycan structures in GlycoCT and IUPAC. However, GlycoCT is not ideally suited for efficient structure similarity search or motif extraction. For these purposes the Resource Description Framework (RDF) scheme is recognized as a preferable option (11, 12). We implemented an RDF data model as the basis of GlyS3 (26), the generic substructure search tool in Glycomics@ExPASy. For example, binding motifs of SugarBindDB are cross-referenced to full structures of GlyConnect using GlyS3.

##### Structure Representation

The 3rd edition of Essentials of Glycobiology, the reference manual in the field, highly recommends the SNFG defined in (16) for representing glycan structures. All the tools in Glycomics@ExPASy are SNFG-compatible. SNFG is also set as the default representation in all SIB databases in which visualization in the Oxford nomenclature (31) and text-IUPAC condensed is optional.

##### Cross-references

All the databases developed in Glycomics@ExPASy describe species with the taxonomy of the National Centre for Biotechnology Information (NCBI) (32) and provide a UniProtKB (33) accession number for each annotated glycoprotein. Furthermore, to facilitate the connection with other glyco-resources and to be compatible with multiple encoding standards, glycan structures are associated with unique identifiers of GlyTouCan (15), the international glycan structure repository. When glycan structures are imported into one of our data sources, we manually check if they are already present in the repository, otherwise we proceed with the registration. The unique identifier is useful to easily connect tools. In addition, GlyTouCan automatically generates cartoons and linear representation in diverse formats.

##### Controled Vocabularies and Ontologies

Although glycan structures are the central elements in the resources of Glycomics@ExPASy, a substantial amount of biological data can be associated with each glycan structure. To ease cross-linking of the information and ensure its quality, we use existing ontologies and controlled vocabularies whenever possible. More specifically, tissue information is systematically tagged with Uberon (34) and Brenda (35) identifiers (now operational in GlyConnect and soon extended to SugarBindDB and UniCarb-DB). Disease associations in SugarBindDB and GlyConnect match appropriate terms from Disease ontology (36).

##### Experimental Evidence

The Minimum Information Required for A Glycomics Experiment (MIRAGE) guidelines have been issued (17) but to date, are not implemented widely. UniCarb-DB, the glycomic mass spectrometry (MS) database and repository (23), is accumulating information in the first MIRAGE-compliant MS-based database (manuscript in preparation).

##### Technical Options for Databases Hosted on ExPASy

The Glycomics@ExPASy backend is built on top of a family of curated databases (Table I). Three (SugarBindDB, UniCarb-DB and Gly Connect) can be directly queried on-line whereas the remainder serve as sources for dedicated tools. To keep both the number of databases and their extent to a minimum, the glyco-epitope collection is made accessible only through applications. As detailed in (37) it results from the compilation of four independent sources (GlycoEpitopeDB, SugarBindDB, Glyco3D (38) and the literature) and the glycoepitopes were all translated into the GlycoCT format. Glycan array binding data were downloaded from the Consortium for Functional Glycomics (CFG) database (http://www.functionalglycomics.org/glycomics/publicdata/) to reinforce the lectin recognition described in SugarBindDB. This data can of course still be queried directly from the CFG website. The *Database* table (Table I) summarizes the data that is fully hosted at SIB. Note that the Unilectin joint project (https://www.unilectin.eu) aiming at classifying and predicting lectins as well as enhance Lectin3D (38) was included in 2018. Data is presently hosted at CERMAV (https://www.cermav.cnrs.fr).

**Table I:** Database table List of databases with data hosted at SIB. Note that Unilectin is developed in collaboration with SIB but data is hosted at CERMAV (https://www.cermav.cnrs.fr).

Status	Name	Purpose	Ref
On-line direct web access	SugarBindDB	Host-pathogen interactions	(24)
	UniCarb-DB	Experimental MS data	(23)
	GlyConnect	Glycoconjugate data (extension of GlycoSuiteDB (48))	−
Only accessed through tool usage	Glycoepitopes	Protein-binding substructures	−
	CFG data	Glycan array experiments	−

All curated data within the Glycomics@ExPASy initiative are stored in relational databases, which form the knowledge base of our websites and tools. The technology stack is similar in our three databases and from a user perspective, the “look and feel” is harmonized. The databases are powered with a very recent version of PostgreSQL (version 9.5) in a dedicated web application for each. These web applications are built with the Java technology and the *Play!* Framework (version 2.5). These options facilitate the development of corresponding Graphical User Interfaces (GUIs) and a REST Application Programming Interface (API) for machine communication.

With the power of the latest version of the database engine, an aggregation of the glycan structures, protein recognition and bindings has led to storing data and annotation in a centralized repository. As a result, a communication Application Programming Interface (API) and GlyConnect acting as a dashboard provide the users with a broader view and the means to query and navigate through the different data sets. Database information is encapsulated in a series of tables, which cannot be accessed from outside SIB. Open access to the data will however be shortly granted via an RDF end point. The end point comes with a new ontology, inspired from GlycoRDF (39) that helps users understanding relationships across different molecular entities. Using the ontology, any computer or user can integrate Glycomics@ExPASy data with external resources that provide a public RDF end point and have an entity in common. The use of RDF and dedicated ontology makes Glycomics@ExPASy compliant with FAIR Guiding Principles for scientific data management (40).

##### Technical Options for In-house Tool Development

The logic behind all Glycomics@ExPASy tools has been shaped using a Service Oriented Architecture (SOA) (41). We implemented each specific task as a service, accessible with a common Application Programming Interface (API), which can be combined to create more complex tools. Our willingness to extend principles of modularity and reusability to the graphical user interface (GUI) prompted the search for a modular framework, which would allow the development of separated GUI components as building blocks in the deployment of new tools. To reach our goal, we followed the Web Component standard created by W3C and Google. It offers an easy composition and reuse of GUI components. As a proof of concept, the framework named Google Polymer was used to redesign the interface of GlycoDigest (previously only available as download (25)). Then it was used more extensively to build SwissMassAbaccus, Glynsight (37), PepSweetener (42), EpitopeXtractor (37) and the Search section of GlyConnect. In the long run, this approach saves time because a new tool can be created with readily available services and GUI building blocks. In addition, the ability to quickly build prototypes based on ideas from the wet lab facilitates collaboration between developers and users.

## RESULTS

ExPASy, now a mature 24-year-old portal accessible to the Life Science community (43), has mainly hosted proteomics tools (44) until 2012 when it was extended to host other -omics resources (22). CAZy (45), GlycanMass and GlycoMod (46) as well as the neural net glyco-site predictor series (47) of the former Center for Biological Sequence Analysis (CBS) (now Bioinformatic unit at Technical University of Denmark) have stood the test of time and portal changes. They preceded the current effort described here to provide access to glyco-resources from ExPASy. This “seed” selection illustrates our willingness to mix in-house with externally developed quality informatic tools in the collection. It also emphasizes the continuity of our endeavor because two authors of GlycoMod (46) are authors of the present article. Note that GlycoSuiteDB (48) was also briefly part of the proteomics collection between 2009 and 2013.

Glycomics@ExPASy is developed in close collaboration with several glycoscience and glycoproteomics groups acknowledged at the end of this article. As such it is destined to expand the range of databases and tools it contains.

### 

#### 

##### In-house Data Collection

Data stored in the listed glycodatabases is in most cases, manually curated. For the SIB-labeled databases, curation is jointly undertaken with glycoscientist partners who supply expert information assessment that defines the filtering and subsequent annotation procedures. However, the expected increase of data production has prompted tagging the stored information as “reviewed” or “unreviewed” depending on the level of data curation. The 21^st^ century is being characterized by a major speed gap between data production and its curation. All -omics fields are undergoing a data deluge to produce the current Big Data challenge. Thus, at this stage “reviewed” glycoprotein information is based on the curated literature based GlycoSuiteDB (48) and further updating work performed by Dr Robyn Peterson in the Packer research group. “Reviewed” glycan mass spectrometry data is annotated by the expert Karlsson group in UniCarb-DB. The “unreviewed” tag usually involves minimal quality check and currently only involved the integration of MS large-scale glycoproteomics data.

The glycan data and information stored in our databases reflects the actual trends in the literature. High-throughput glycopeptide studies as illustrated in the latest published works (49, 50) are overwhelmingly focused on human *N*-glycosylation. These large data sets are now included in GlyConnect with an “unreviewed” status. Despite this subsequent and temporary bias toward human *N*-glycans reflecting data availability, GlyConnect content is diverse and cross-species. It does cover a significant number of *O*-glycans. In contrast, UniCarb-DB is biased toward *O*-glycan spectra. A greater integration of these two databases is planned in the near future. GlyConnect also contains a few entries of C-linked glycans which are much rarer in the literature as well as structures of glycans not attached to proteins such as milk oligosaccharides.

UniLectin was launched in 2018 and currently contains an extended version of the Lectin3D part of the Glyco3D collection (38). It is dedicated to describing lectins and their glycan ligands initially based on information stored in the PDB (Protein Data Bank). Another (yet unpublished) aspect of the UniLectin project is to predict lectin domains and folds in uncharacterized amino acid sequences.

##### Selection of External Data Sources

Because of the growing interest in glycomics, several glycoscience groups around the world are releasing new or updated versions of glycoinformatics tools and databases. Glycomics@ExPASy is set to integrate strictly web-based, quality recognized and supported resources for glycomics. Tools and databases developed by our group at SIB are preceded by the SIB logo whereas external resources have an “external link” gray icon.

The inclusion of MatrixDB (51) and CSDB (52) in the database list of Glycomics@ExPASy offers access to curated glycosaminoglycans (GAGs) and bacterial and fungal glycan information respectively. The registration in the GlyTouCan repository of all glycan structures recorded in our own and in partner databases greatly facilitates integrative efforts. This task has required substantial preliminary work on data standardization and was carried out for GAGs in collaboration with MatrixDB developers (manuscript resubmitted after minor revision).

The duplication of the link to Glycomics@ExPASy of the renowned CAZy database of glycosylation enzymes from the ExPASy proteomics tab, where it has been listed for many years, improves the set of relevant databases by covering the knowledge of glycan synthesis and degradation.

External resources are listed in supplemental Table S1. In the vast majority of cases, they have been selected either because they are cross-referenced in our resources (this is the case for example of GlyTouCan or Glyco3D), or are planned to be in the near future. Our latest addition to the Database section is Glycopedia that provides a source of basic to advanced knowledge of glycoscience. Glycopedia collects e-chapters and compiles various sources to suggest relevant readings. It is consulted by naïve or expert users and as such is increasingly referred to in and outside the field of glycoscience.

We include resources upon suggestion or request. Note that as a rule, applying to most sections of ExPASy, to be included in the portal, the resources must be web-based, regularly updated and improved. With these criteria met, we are open to grow the collection with those qualifying resources. Our contact form on the ExPASy website is suited for suggesting a tool or a database to be assessed and added. The inclusion process is ongoing.

##### Dashboard

**Fig. 2.**
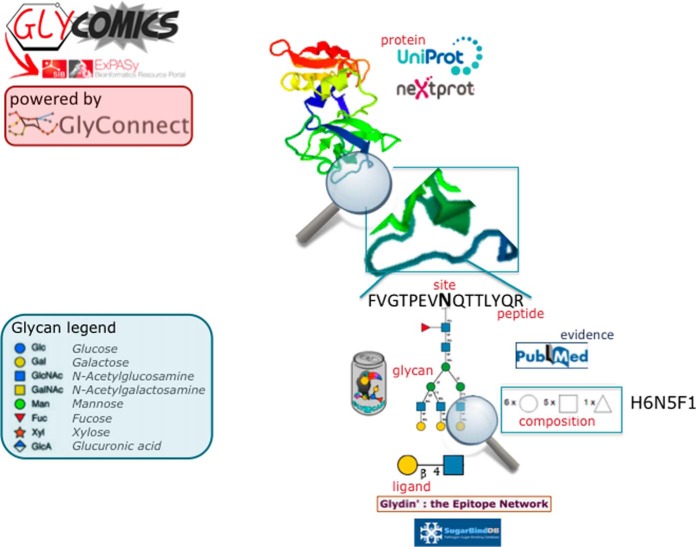
**The essential entities described in the resources of Glycomics@ExPASy.** One of the main purposes of Glycomics@ExPASy is the integration of the tools in the collection shown in Fig. 1. To that end, GlyConnect is used as a dashboard for using in-house and cross-referenced resources. It is designed to ease navigation between entities named in red: protein, peptide, site, glycan, composition and ligand. A protein, a glycan composition or a glycan structure (through its structural properties) is an entry point in the database. Then structures, peptides and sites can be listed and compared and possible correlations brought out.

We briefly introduce here GlyConnect, our new dashboard released in December 2017 and unpublished so far. GlyConnect is designed to monitor, integrate and facilitate the interpretation of collected glycomics and glycoproteomics data. It is the central platform of Glycomics@ExPASy that will boost the usefulness of on-line services by tightly integrating tools and databases. The essential entities that are stored or processed in GlyConnect, are shown in Fig. 2.

##### Integrative Tools

Database associated tools can be grouped into two categories, either *dedicated* to solve a specific question or *integrative* to be used in several applications. The collection is depicted in this way in Table II and III. Most dedicated tools are described in detail in the cited references. For example, integrative tools are:

- Substructure search, GlyS3 is used in linking SugarBindDB to GlyConnect, in the construction of the Glydin' map and in some queries of GlycoSiteAlign,
- EpitopeXtractor is integrated in Glynsight and GlyConnect,
- GlycanBuilder, developed within the EuroCarb project (53) is used as a query tool in the databases GlycoDigest and GlyS3. It will be soon replaced by SugarSketcher, a new web component currently being prototyped. The beta version is nonetheless accessible on the server (https://glycoproteome.expasy.org/sugarsketcher) and awaits feedback from users,
- LiteMol (54) was also not developed in our group but is used across our resources for visualizing 3D structures when available in the PDB (55). LiteMol was selected as the main 3D visualizer in the PDBe, the European version of PDB World.

**Table II:** Dedicated tool table This table lists all the tools developed by SIB. For each tool the table shows a short description followed by its reference. In addition, we report the software language that has been used and if the tool is accessed through a web interface written with Google Polymer web components.

Name	Purpose	Ref	Web component	Language
GlycoMod	Predicts glycan structures from mass data	(46)	No	Perl
GlycoDigest	Simulates exoglycosidase digestion	(25)	Yes	Javascript
SwissMassAbacus	Glycopeptide mass calculator	−	Yes	Javascript
GlycoSiteAlign	Aligns glycosite regions depending on attached glycan	(27)	No	Python
PepSweetener	Predicts intact glycopeptides (peptide + glycan composition) from mass data	(42)	Yes	Javascript
Glycoforest	Partial de novo sequencing of glycans from MS/MS data	(60)	No	Java
Glynsight	Displays interactively glycan profile changes on a single protein in multiple conditions	(37)	Yes	Javascript

**Table III:** Integrative tool table This table lists all the integrative tools developed by SIB. For each tool the table shows a short description followed by its reference. In addition, we report the software language that has been used and if the tool is accessed through a web interface written with Google Polymer web components. Rows written in grey correspond to software developed outside SIB.

Name	Purpose	Ref	Web component	Language
GlycanBuilder	Graphic interface for drawing glycan (operational but slow)	(53)	No	Java
SugarSketcher	Client-side graphic interface for drawing glycan (prototype)	−	Yes	Javascript
Glydin'	Displays interactively a map of glycoepitopes and shared substructures	(37)	No	Python
GlyS3	Glycan substructure search	(26)	Yes	Java
EpitopeExtractor	Outputs known glycan determinants from glycan structures	(37)	Yes	Javascript
LiteMol	Displays interactively 3D models of glycoproteins	(54)	No	Javascript

##### Hypothesis Building

Glycomics@ExPASy attempts to support the formulation of hypothese in the broad range of biological functions where glycans are involved. We offer a toolbox to navigate, explore and correlate data. As an example, using GlyS3, SugarBindDB, GlyConnect and the respective cross-links of these databases to UniProt, a user can seek to establish the consistency of interactions taking place at the cell surface. In the following, three possible use cases are brought to the reader.

##### From MS to Glycoprotein Features

**Fig. 3.**
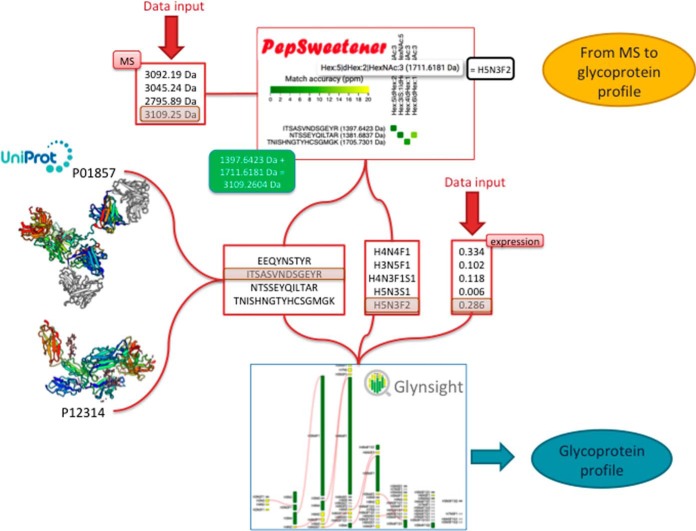
**From Mass Spectrometry data to glycoprotein profile.** A representative scenario of the possible combination of PepSweetener and Glynsight in order to support the manual annotation of MS1 mass spectra of intact *N*-glycopeptides and integrate quantitative information when available. Users can process MS1 Spectra using PepSweetener to identify all the possible *N*-glycan compositions on a single human protein. Intact glycopeptide masses are broken into the respective contributions of the peptide and the glycan masses. Compositions in PepSweetener are in the detailed format shown in supplemental Fig. S1. Then, when quantitative data on each composition is available, Glynsight can be used to identify specific glycosylation patterns. The procedure can be repeated with a second protein and Glynsight will automatically generate the differential analysis of glycan profiles on the proteins. The integration with Glyconnect leads to displaying the potential glycan structures known to match the differentially expressed monosaccharide compositions.

Our tool set is designed to match the expected boost of glycoproteomics (glycan composition at specific sites on complex mixtures of glycoproteins) data that is currently just reaching high throughput level (56, 57). An example on how to integrate some of our dedicated tools for extracting glycoprotein features from MS data is shown in Fig. 3. Predominant precursor masses in the MS spectra can be input into PepSweetener (42). This software supports the manual annotation of intact glycopeptides, using custom web visualization regardless of the instrument that produced the data. Results are displayed on an interactive heat-map chart featuring the combined mass contributions of theoretical (usually tryptic) peptides and attached glycan compositions. The variations in tile colors correspond to ppm deviations from the query precursor mass. Annotation can be refined through glycan composition filtering, sorting by mass and tolerance, and checking MS2 data consistency via an *in silico* peptide fragmentation diagram (in-house fragmentation tool common with that of UniCarb-DB). PepSweetener is mainly designed as a complement or extension to software being developed for automatic analysis of glycoproteomics MS data and avoiding their dependence on a set workflow or type of instrument (13). Note that an international assessment of the latter tools available for the interpretation of complex glycopeptide MS/MS data is underway through a call launched in 2018 within the HUPO Human Glycoproteomics Initiative (HGI: https://www.hupo.org/Glycoproteomics-(B/d-GPP)) for testing the performance of the currently available glycoproteomics MS software with benchmark data sets. The outcome of this study will guide the presentation of the Glycomics@ExPASy toolbox toward a more informative and didactic section on MS-based glycoproteomics data analysis tools. Given that we collect exclusively web-based resources rules out the inclusion of efficient but only stand-alone software. One such web-based proteomics viewer caters for the visualization of glycoproteomics MS data, namely MSViewer, integrated in the Protein Prospector software suite that uses a specific glycosite database to enhance intact glycopeptide identification (58). Protein Prospector features in the proteomics tab of the ExPASy portal as it is currently recognized as a protein identification platform.

In the best cases, glycoproteomics experiments also generate relative quantitative data (see (59) for example). In such favorable, though presently rare, data, a set of site-specific glycan compositions can be associated with levels of abundance, which can then be processed by Glynsight that will create glycan profiles for each glycoprotein in the experiment (37). A file containing a list of glycan compositions present on a protein, with the respective quantification, can be uploaded and processed by Glynsight. This tool produces a custom visualization that highlights up/down-regulated glycan compositions (either site-specific or global) among diverse proteins or on the same protein under different conditions, for example healthy/cancer. Fig. 3 highlights the situation where two proteins are successively processed with PepSweetener and assumes quantitative data are provided for each identified glycan composition. The Glynsight interface can display a glycan expression profile for each protein as well as the differential profile between two proteins. Furthermore, Glynsight integrates with GlyConnect, such that any glycan composition is connected to all the putative glycan structures that have already been reported in the literature. In the end, after determining a pool of interesting compositions from MS data, scientists can leverage knowledge and tools in Glycomics@ExPASy to build new hypotheses and design the next round of experiments.

##### Exploring Glycoprotein Features

**Fig. 4.**
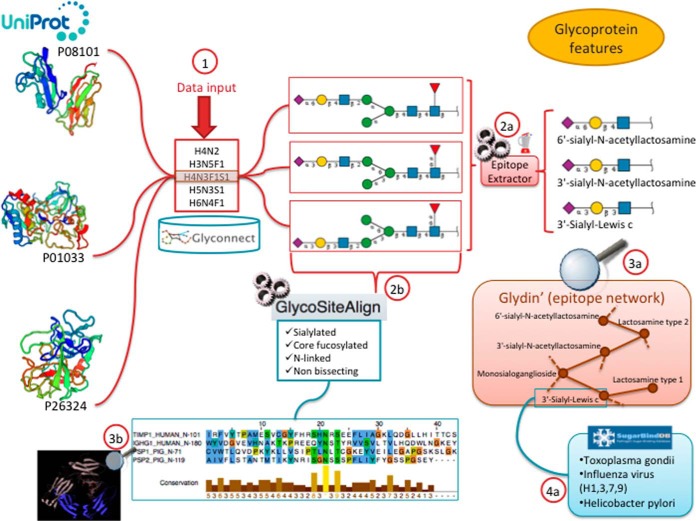
**From composition to glycoprotein features.** An interactive way of extracting glycoprotein features from glycan compositions combining published data and *ad hoc* tools (1). A list of compositions is input in GlyConnect, which retrieves all the proteins reported as having these compositions attached to them (on the left) and reported glycan structures corresponding to this composition (on the right) annotated in this knowledge base. Glycan structures can be further processed to extract contained glycan epitopes using EpitopeXtractor (2a). Glycoepitope results can be mapped on Glydin', an interactive epitope network (3a). Glydin' aggregates glycan epitopes from four different sources (databases and literature reviews) and provides links to the original information. When epitopes are taken from SugarBindDB, further information on the pathogens can be browsed (4a).

Current global glycome profiling experiments generate one or more set(s) of glycan compositions and/or structures with their respective expression on a protein, in a tissue or in a cell. Tools and databases in Glycomics@ExPASy can be combined to explore distinctive glycan features that characterize glycoproteins as shown in Fig. 3. In this case, the entry point of the workflow is GlyConnect to which a list of glycan compositions is submitted. The GlyConnect search tool will retrieve the possible related glycan structures and the proteins that have been reported to have these compositions/structures attached and stored in the databases. Results are displayed in a form of a conceptual map where the compositions sit in the middle and connect glycan structures and associated glycoproteins, respectively on the right and the left sides (Fig. 4 part 1). This visualization is well suited for understanding the potential relations between proteins and glycans. Selected glycan structures can be further explored through activating the integrated EpitopeXtractor function (Fig. 4 part 2a). This tool extracts all glycoepitopes that form a part of the selected structures, based on our curated set of glycoepitopes (see Material & Methods section). To further explore the results, Epitope Xtractor is integrated with Glydin' (37) (Fig. 4 part 3a), the epitope network viewer so that extracted glycoepitopes can be mapped onto the network to highlight clusters of shared structures as well as potential outliers. Glydin' provides two types of network: substructure based and enzyme based. In the substructure case two nodes are connected if one glycoepitope is a substructure of the other whereas in the enzyme case, two nodes are connected as the result of a glycosyltransferase adding a monosaccharide to the other. Each node/glycoepitope, is linked to the database or publication source(s) from which it was extracted (see (37) for details). When glycoepitopes are contained in SugarBindDB (Fig. 4 part 4a), additional information includes structures potentially associated with diseases.

This stepwise combination of tools draws a possible path from a list of glycan compositions to the binding properties of specific glycan structures to support the user in narrowing down questions of molecular interaction and selecting a subset of relevant glycans. The structural properties of these interacting glycan structures can be further exploited. In both GlyConnect and GlycoSiteAlign (27) glycan structures are associated with descriptive features of glycan type such as “sialylated”, “core-fucosylated”, etc (see supplemental Table S2 for the complete set of properties). In parallel to considering the set of glycan structures matching the initial list of compositions as the input of EpitopeXtractor, the compositions can also be submitted to GlycoSiteAlign. This tool selectively aligns amino acid sequences surrounding glycosylation sites (by default, 20 positions on each side of the glycosylated residue) depending on structural properties of the known glycan attached to the site. In other words, this is an alternative mean to characterize the glyco-site sequence environment with the prospect of identifying the most constrained amino acids.

##### Glycan-mediated Protein-Protein Interactions

**Fig. 5.**
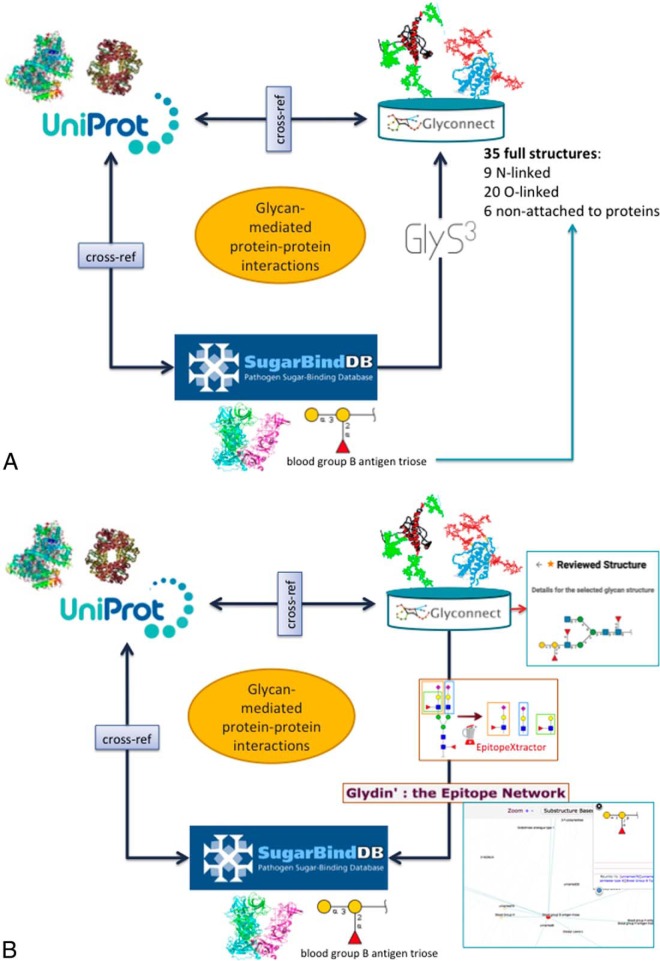
**Glycan mediated protein-protein interactions.** This figure shows how a new hypothesis on glycan mediated protein-protein interaction can be built using published data in Glyconnect and SugarBindDB: *A*, In this scenario glycan binding protein (GBP) is selected in SugarBind. The information on the glycan ligand recognized by the GBP is used to perform a substructure search on all the structures in Glyconnect with the GlyS3 glycan substructure search tool. The structures identified by GlyS3 are used in Glyconnect to create a list of target proteins that can interact with the initial GBP. In the example of the blood group B antigen triose, there are 35 full structure types in GlyConnect that contain this glycoepitope (*B*) In this scenario a glycoprotein in GlyConnect is selected with its list of associated glycan structures. Glycans are processed with EpitopeXtractor to single out all the glycan epitopes contained. The Glydin' interactive map of structurally related glycoepitopes helps visualizing the potential common substructures in the complete set of glycoepitopes. Then, extracted epitopes are used in SugarBind to identify all the reported GBPs that can possibly interact with the initial glycoprotein. In this example, the VP1 capsid protein of the Norwalk virus is known to bind the blood group B antigen triose. Note that protein structures shown above UniProt and those shown above GlyConnect are not related to the example but simply illustrating the difference in the information that is stored on the unglycosylated protein in contrast with the stored information on intact glycoproteins.

Using another combination of tools and databases in Glycomics@ExPASy, potential correlations between a glycan binding protein (GBP) of a pathogen a host glycoprotein and a glycan structure (Fig. 5*B*) can be made.

In the first scenario (Fig. 5*A*), the starting point is a glycoepitope recognized by a specific GBP (Fig. 5*A*), a bacterial lectin described in SugarBindDB. This is illustrated with the blood group B antigen triose. A binding event in this database is always formed by a pair composed of a GBP/lectin and a glycoepitope part of a glycan present on the host surface. Whenever possible, further information of a GBP/lectin is available via cross-reference to UniProt. The glycoepitope can be used as an input of the GlyS3 substructure search tool to match the full structures stored in GlyConnect that contain this specific ligand. The list of glycan structures retrieved by GlyS3 can be explored in GlyConnect that reports relationships between glycans and glycoproteins. In this example 35 matches to full structures are detailed. They are spread across twenty *O*-linked, nine *N*-linked glycans and six structures reported as non-attached to proteins. The shortlisted glycoproteins can be considered as candidates for interacting with the initial GBP/lectin. The cross-references from GlyConnect to UniProt closes the loop for protein information. In a nutshell, starting with a GBP/lectin can lead to a selection of putative glycoprotein interaction partners.

The second scenario (Fig. 5*B*) starts from a glycan structure in GlyConnect and relies on its reported relationships with glycoproteins. The figure shows an example of a reviewed *N*-linked glycan structure. GlyConnect also offers the option of running EpitopeXtractor to generate a selection of glycoepitopes contained in this starting glycan. Leveraging the binding data in SugarBindDB, the obtained glycoepitopes can be associated with a collection of GBPs/lectins that recognize one or more of these glycoepitopes. In the end, the workflow allows the selection of GBPs that could possibly interact with the glycoproteins on which the starting glycan has been reported to be attached. Cross-references of both glycoproteins in GlyConnect and GBPs/lectins in SugarBindDB to UniProt can be used to further rationalize potential interacting partners.

In the end, combinations of tools and databases in Glycomics@ExPASy can be used to generate a selection of putative glycan mediated protein-protein interactions. These can be considered for further experiments.

## DISCUSSION

Our project is part of a wider initiative and is intended as a major step toward interconnecting isolated efforts within the broad and interdisciplinary field of glycobiology. As such it is designed to boost progress in glycoscience research. At this stage, we have a focus on human glycans and related proteins reflecting the current main research published in the literature. Cell surfaces, plasma and serum are the most studied systems displaying glycosylation changes in many diseases. However, there is overwhelming evidence of similar changes on other glycoproteins, components of the immune system, saliva and the protective mucus of the gastrointestinal tract, among others. Many of these glycosylation changes are biologically significant and connected with the molecular dysfunctions involved in disease initiation and are evident early in the disease progress. Being key mediators in tumor progression events, glycoproteins influence features of tumor cells such as proliferation, invasion, angiogenesis, and metastasis. Besides, glycans and glycoconjugates play essential roles in the dynamic interplay between host and pathogens.

Our technical choices detailed in the Material & Methods section were deliberately made to ensure the modularity, interoperability, reusability and user-friendliness of our applications on the ExPASy portal. In the long run, this approach saves time because a new tool can be created with readily available services and Graphical User Interface (GUI) building blocks. To give a concrete example, SwissMassAbacus was prototyped in 1 day, assembling available pieces and quickly deployed within a week. In addition, the ability to build quick prototypes based on ideas from the wet lab facilitates the collaboration between developers and users. The innovative component of this approach is not only related with time but has an impact on work sharing. The modularity enforced by the framework allows assigning precise and delimited tasks that can be undertaken by software developers with no prior involvement in the project. Web Components and SOA are key to the fast delivery of *ad hoc* applications to our end users mixing pre-built GUI components with a solid API. Maintenance is minimal and easily transferred to new staff. Finally, our tight network with glycoscientists contributes to biocuration and the definition of tool requirements and is creating a critical mass of users and the best conditions for anticipating the bioinformatics needs of the community. This privileged position combined with rationally chosen technology seems our best chance to guarantee long term production, quality, usefulness and maintenance.

We are aware that the scenarios we have proposed to combine several tools and rely on curated data are still limited by the minimal content of available databases. As an example, we are collecting data from published glycoproteomics data sets including the increasing *O*-GlcNAc data. We anticipate this shortcoming of limited data to lessen with time with converging efforts to produce more glycoproteomics data and improved experimental technologies. Glycomics@ExPASy has been designed to accommodate this easily.

## CONCLUSION

Glycomics@ExPASy is destined to become an essential and valuable reference portal for glycoscience research. Offering bioinformatics resources for glycomics and glycoproteomics on a server that has built its long-lasting reputation on bioinformatics for proteomics and more recently for other common -omics is an effective means of pulling glycoscience out of its isolation by boosting visibility and cross disciplinary research focus. Glycomics@ExPASy was designed and is developed with a long-term vision and in collaboration with glycoscientists to meet as closely as possible the needs of the community in glycoinformatics. This vision in the first instance encompasses reaching out to protein scientists who consider glycomics a too complicated topic to include in protein characterization studies. Next steps will involve the inclusion of data on other biologically important glycoconjugate data such as glycolipids and proteoglycans and their connection with other -omics data.

## Supplementary Material

supplemental Table S1

## References

[1] National Research Council (US) Committee on Assessing the Importance and Impact of Glycomics and Glycosciences. (2012) Transforming Glycoscience: A Roadmap for the Future (National Academies Press (US), Washington (DC)) 23270009

[2] Wuhrer M. (2013) Glycomics using mass spectrometry. Glycoconj. J. 30, 11–22 22532006 10.1007/s10719-012-9376-3 PMC3547245

[3] Lundborg M., and Widmalm G. (2011) Structural analysis of glycans by NMR chemical shift prediction. Anal. Chem. 83, 1514–1517 21280662 10.1021/ac1032534

[4] Adamczyk B., Stöckmann H., O'Flaherty R., Karlsson N. G., and Rudd P. M. (2017) in High-Throughput Glycomics and Glycoproteomics, eds Lauc G, Wuhrer M (Springer New York, New York, NY), pp 97–108

[5] Ruhaak L. R., Hennig R., Huhn C., Borowiak M., Dolhain R. J. E. M., Deelder A. M., Rapp E., and Wuhrer M. (2010) Optimized workflow for preparation of APTS-labeled N-glycans allowing high-throughput analysis of human plasma glycomes using 48-channel multiplexed CGE-LIF. J. Proteome Res. 9, 6655–6664 20886907 10.1021/pr100802f

[6] Grant O. C., and Woods R. J. (2014) Recent advances in employing molecular modelling to determine the specificity of glycan-binding proteins. Curr. Opin. Struct. Biol. 28, 47–55 25108191 10.1016/j.sbi.2014.07.001 PMC4252743

[7] Cecioni S., Praly J.-P., Matthews S. E., Wimmerová M., Imberty A., and Vidal S. (2012) Rational design and synthesis of optimized glycoclusters for multivalent lectin-carbohydrate interactions: influence of the linker arm. Chem. - Eur. J. 18, 6250–6263 22488581 10.1002/chem.201200010

[8] Pilobello K. T., Krishnamoorthy L., Slawek D., and Mahal L. K. (2005) Development of a lectin microarray for the rapid analysis of protein glycopatterns. ChemBioChem 6, 985–989 15798991 10.1002/cbic.200400403

[9] Heimburg-Molinaro J., Song X., Smith D. F., and Cummings R. D. (2011) in Curr. Protoc. Protein Sci., eds Coligan JE, Dunn BM, Speicher DW, Wingfield PT (John Wiley & Sons, Inc., Hoboken, NJ, U.S.A.), p 12.10.1–12.10.29

[10] Thaysen-Andersen M., Packer N. H., and Schulz B. L. (2016) Maturing glycoproteomics technologies provide unique structural insights into the *N*-glycoproteome and its regulation in health and disease. Mol. Cell Proteomics 15, 1773–1790 26929216 10.1074/mcp.O115.057638 PMC5083109

[11] Campbell M. P., Ranzinger R., Lütteke T., Mariethoz J., Hayes C. A., Zhang J., Akune Y., Aoki-Kinoshita K. F., Damerell D., Carta G., York W. S., Haslam S. M., Narimatsu H., Rudd P. M., Karlsson N. G., Packer N. H., and Lisacek F. (2014) Toolboxes for a standardised and systematic study of glycans. BMC Bioinformatics 15, S9 10.1186/1471-2105-15-S1-S9 PMC4016020 24564482

[12] Campbell M. P., Aoki-Kinoshita K. F., Lisacek F., York W. S., and Packer N. H. (2015) in Essentials of Glycobiology, eds Varki A, Cummings RD, Esko JD, Stanley P, Hart GW, Aebi M, Darvill AG, Kinoshita T, Packer NH, Prestegard JH, Schnaar RL, Seeberger PH (Cold Spring Harbor Laboratory Press, Cold Spring Harbor (NY)). 3rd Ed. 27010055

[13] Walsh I., O'Flaherty R., and Rudd P. M. (2017) Bioinformatics applications to aid high-throughput glycan profiling. Perspect. Sci. 11, 31–39

[14] Hu H., Khatri K., and Zaia J. (2016) Algorithms and design strategies towards automated glycoproteomics analysis: algorithms and design strategies. Mass Spectrom. Rev. 36, 475–498 26728195 10.1002/mas.21487 PMC4931994

[15] Tiemeyer M., Aoki K., Paulson J., Cummings R. D., York W. S., Karlsson N. G., Lisacek F., Packer N. H., Campbell M. P., Aoki N. P., Fujita A., Matsubara M., Shinmachi D., Tsuchiya S., Yamada I., Pierce M., Ranzinger R., Narimatsu H., and Aoki-Kinoshita K. F. (2017) GlyTouCan: an accessible glycan structure repository. Glycobiology 27, 915–919 28922742 10.1093/glycob/cwx066 PMC5881658

[16] Varki A., Cummings R. D., Aebi M., Packer N. H., Seeberger P. H., Esko J. D., Stanley P., Hart G., Darvill A., Kinoshita T., Prestegard J. J., Schnaar R. L., Freeze H. H., Marth J. D., Bertozzi C. R., Etzler M. E., Frank M., Vliegenthart J. F., Lütteke T., Perez S., Bolton E., Rudd P., Paulson J., Kanehisa M., Toukach P., Aoki-Kinoshita K. F., Dell A., Narimatsu H., York W., Taniguchi N., and Kornfeld S. (2015) Symbol nomenclature for graphical representations of glycans. Glycobiology 25, 1323–1324 26543186 10.1093/glycob/cwv091 PMC4643639

[17] York W. S., Agravat S., Aoki-Kinoshita K. F., McBride R., Campbell M. P., Costello C. E., Dell A., Feizi T., Haslam S. M., Karlsson N., Khoo K.-H., Kolarich D., Liu Y., Novotny M., Packer N. H., Paulson J. C., Rapp E., Ranzinger R., Rudd P. M., Smith D. F., Struwe W. B., Tiemeyer M., Wells L., Zaia J., and Kettner C. (2014) MIRAGE: The minimum information required for a glycomics experiment. Glycobiology 24, 402–406 24653214 10.1093/glycob/cwu018 PMC3976285

[18] Kolarich D., Rapp E., Struwe W. B., Haslam S. M., Zaia J., McBride R., Agravat S., Campbell M. P., Kato M., Ranzinger R., Kettner C., and York W. S. (2013) The minimum information required for a glycomics experiment (MIRAGE) project: improving the standards for reporting mass-spectrometry-based glycoanalytic Data. Mol. Cell. Proteomics 12, 991–995 23378518 10.1074/mcp.O112.026492 PMC3617344

[19] Lisacek F., Mariethoz J., Alocci D., Rudd P. M., Abrahams J. L., Campbell M. P., Packer N. H., Ståhle J., Widmalm G., Mullen E., Adamczyk B., Rojas-Macias M. A., Jin C., and Karlsson N. G. (2017) in High-Throughput Glycomics and Glycoproteomics, eds Lauc G, Wuhrer M (Springer New York, New York, NY), pp 235–264 10.1007/978-1-4939-6493-2_18 27743371

[20] Lutteke T. (2006) GLYCOSCIENCES.de: an Internet portal to support glycomics and glycobiology research. Glycobiology 16, 71R–81R 10.1093/glycob/cwj049 16239495

[21] Akune Y., Hosoda M., Kaiya S., Shinmachi D., and Aoki-Kinoshita K. F. (2010) The RINGS resource for glycome informatics analysis and data mining on the Web. OMICS J. Integr. Biol. 14, 475–486 10.1089/omi.2009.0129 20726803

[22] Artimo P., Jonnalagedda M., Arnold K., Baratin D., Csardi G., de Castro E., Duvaud S., Flegel V., Fortier A., Gasteiger E., Grosdidier A., Hernandez C., Ioannidis V., Kuznetsov D., Liechti R., Moretti S., Mostaguir K., Redaschi N., Rossier G., Xenarios I., and Stockinger H. (2012) ExPASy: SIB bioinformatics resource portal. Nucleic Acids Res. 40, W597–W603 22661580 10.1093/nar/gks400 PMC3394269

[23] Hayes C. A., Karlsson N. G., Struwe W. B., Lisacek F., Rudd P. M., Packer N. H., and Campbell M. P. (2011) UniCarb-DB: a database resource for glycomic discovery. Bioinforma. Oxf. Engl. 27, 1343–1344 10.1093/bioinformatics/btr137 21398669

[24] Mariethoz J., Khatib K., Alocci D., Campbell M. P., Karlsson N. G., Packer N. H., Mullen E. H., and Lisacek F. (2016) SugarBindDB, a resource of glycan-mediated host–pathogen interactions. Nucleic Acids Res. 44, D1243–D1250 26578555 10.1093/nar/gkv1247 PMC4702881

[25] Gotz L., Abrahams J. L., Mariethoz J., Rudd P. M., Karlsson N. G., Packer N. H., Campbell M. P., and Lisacek F. (2014) GlycoDigest: a tool for the targeted use of exoglycosidase digestions in glycan structure determination. Bioinformatics 30, 3131–3133 25015990 10.1093/bioinformatics/btu425 PMC4609004

[26] Alocci D., Mariethoz J., Horlacher O., Bolleman J. T., Campbell M. P., and Lisacek F. (2015) Property Graph vs RDF Triple Store: A comparison on glycan substructure search. PLOS ONE 10, e0144578 26656740 10.1371/journal.pone.0144578 PMC4684231

[27] Gastaldello A., Alocci D., Baeriswyl J.-L., Mariethoz J., and Lisacek F. (2016) GlycoSiteAlign: glycosite alignment based on glycan structure. J. Proteome Res. 15, 3916–3928 27523326 10.1021/acs.jproteome.6b00481

[28] Varki A. (2017) New and updated glycoscience-related resources at NCBI. Glycobiology 27, 993–993 28973256 10.1093/glycob/cwx077

[29] Horlacher O., Nikitin F., Alocci D., Mariethoz J., Müller M., and Lisacek F. (2015) MzJava: An open source library for mass spectrometry data processing. J. Proteomics 129, 63–70 26141507 10.1016/j.jprot.2015.06.013

[30] Herget S., Ranzinger R., Maass K., and Lieth C.-W. (2008) v. d. GlycoCT—a unifying sequence format for carbohydrates. Carbohydr. Res. 343, 2162–2171 18436199 10.1016/j.carres.2008.03.011

[31] Harvey D. J., Merry A. H., Royle L., Campbell M. P., Dwek R. A., and Rudd P. M. (2009) Proposal for a standard system for drawing structural diagrams of N- and O-linked carbohydrates and related compounds. Proteomics 9, 3796–3801 19670245 10.1002/pmic.200900096

[32] Federhen S. (2012) The NCBI Taxonomy database. Nucleic Acids Res. 40, D136–D143 22139910 10.1093/nar/gkr1178 PMC3245000

[33] UniProt Consortium, T. (2018) UniProt: the universal protein knowledgebase. Nucleic Acids Res. 46, 2699–2699 29425356 10.1093/nar/gky092 PMC5861450

[34] Mungall C. J., Torniai C., Gkoutos G. V., Lewis S. E., and Haendel M. A. (2012) Uberon, an integrative multi-species anatomy ontology. Genome Biol. 13, R5 22293552 10.1186/gb-2012-13-1-r5 PMC3334586

[35] Gremse M., Chang A., Schomburg I., Grote A., Scheer M., Ebeling C., and Schomburg D. (2011) The BRENDA Tissue Ontology (BTO): the first all-integrating ontology of all organisms for enzyme sources. Nucleic Acids Res. 39, D507–D513 21030441 10.1093/nar/gkq968 PMC3013802

[36] Schriml L. M., Arze C., Nadendla S., Chang Y.-W. W., Mazaitis M., Felix V., Feng G., and Kibbe W. A. (2012) Disease Ontology: a backbone for disease semantic integration. Nucleic Acids Res. 40, D940–D946 22080554 10.1093/nar/gkr972 PMC3245088

[37] Alocci D., Ghraichy M., Barletta E., Gastaldello A., Mariethoz J., and Lisacek F. (2018) Understanding the glycome: an interactive view of glycosylation from glycocompositions to glycoepitopes. Glycobiology 28, 349–362 29518231 10.1093/glycob/cwy019

[38] Pérez S., Sarkar A., Rivet A., Breton C., and Imberty A. (2015) in Glycoinformatics, eds Lütteke T, Frank M (Springer New York, New York, NY), pp 241–258

[39] Ranzinger R., Aoki-Kinoshita K. F., Campbell M. P., Kawano S., Lutteke T., Okuda S., Shinmachi D., Shikanai T., Sawaki H., Toukach P., Matsubara M., Yamada I., and Narimatsu H. (2015) GlycoRDF: an ontology to standardize glycomics data in RDF. Bioinformatics 31, 919–925 25388145 10.1093/bioinformatics/btu732 PMC4380026

[40] Wilkinson M. D., Dumontier M., Aalbersberg Ij Appleton J. G., Axton M., Baak A., Blomberg N., Boiten J.-W., da Silva Santos L. B., Bourne P. E., Bouwman J., Brookes A. J., Clark T., Crosas M., Dillo I., Dumon O., Edmunds S., Evelo C. T., Finkers R., Gonzalez-Beltran A., Gray A. J. G., Groth P., Goble C., Grethe J. S., Heringa J., 't Hoen P. A., Hooft R., Kuhn T., Kok R., Kok J., Lusher S. J., Martone M. E., Mons A., Packer A. L., Persson B., Rocca-Serra P., Roos M., van Schaik R., Sansone S.-A., Schultes E., Sengstag T., Slater T., Strawn G., Swertz M. A., Thompson M., van der Lei J., van Mulligen E., Velterop J., Waagmeester A., Wittenburg P., Wolstencroft K., Zhao J., and Mons B. (2016) The FAIR Guiding Principles for scientific data management and stewardship. Sci. Data 3, 160018 26978244 10.1038/sdata.2016.18 PMC4792175

[41] Laskey K. B., and Laskey K. (2009) Service oriented architecture: Service oriented architecture. Wiley Interdiscip. Rev. Comput. Stat. 1, 101–105

[42] Domagalski M. J., Alocci D., Almeida A., Kolarich D., and Lisacek F. (2017) PepSweetener: A Web-based tool to support manual annotation of intact glycopeptide MS spectra. PROTEOMICS - Clin. Appl. 1700069 10.1002/prca.201700069 28975713

[43] Appel R. D., Bairoch A., and Hochstrasser D. F. (1994) A new generation of information retrieval tools for biologists: the example of the ExPASy WWW server. Trends Biochem. Sci. 19, 258–260 8073505 10.1016/0968-0004(94)90153-8

[44] Gasteiger E., Gattiker A., Hoogland C., Ivanyi I., Appel R. D., and Bairoch A. (2003) ExPASy: The proteomics server for in-depth protein knowledge and analysis. Nucleic Acids Res. 31, 3784–3788 12824418 10.1093/nar/gkg563 PMC168970

[45] Lombard V., Golaconda Ramulu H., Drula E., Coutinho P. M., and Henrissat B. (2014) The carbohydrate-active enzymes database (CAZy) in 2013. Nucleic Acids Res. 42, D490–D495 24270786 10.1093/nar/gkt1178 PMC3965031

[46] Cooper C. A., Gasteiger E., and Packer N. H. (2001) GlycoMod–a software tool for determining glycosylation compositions from mass spectrometric data. Proteomics 1, 340–349 11680880 10.1002/1615-9861(200102)1:2<340::AID-PROT340>3.0.CO;2-B

[47] Blom N., Sicheritz-Pontén T., Gupta R., Gammeltoft S., and Brunak S. (2004) Prediction of post-translational glycosylation and phosphorylation of proteins from the amino acid sequence. Proteomics 4, 1633–1649 15174133 10.1002/pmic.200300771

[48] Cooper C. A., Joshi H. J., Harrison M. J., Wilkins M. R., and Packer N. H. (2003) GlycoSuiteDB: a curated relational database of glycoprotein glycan structures and their biological sources. 2003 update. Nucleic Acids Res. 31, 511–513 12520065 10.1093/nar/gkg099 PMC165546

[49] Bollineni R. C., Koehler C. J., Gislefoss R. E., Anonsen J. H., and Thiede B. (2018) Large-scale intact glycopeptide identification by Mascot database search. Sci. Rep. 8 10.1038/s41598-018-20331-2 PMC5795011 29391424

[50] Hu Y., Shah P., Clark D. J., Ao M., and Zhang H. (2018) Reanalysis of global proteomic and phosphoproteomic data identified a large number of glycopeptides. Anal. Chem. 90, 8065–8071 29741879 10.1021/acs.analchem.8b01137 PMC6440470

[51] Launay G., Salza R., Multedo D., Thierry-Mieg N., and Ricard-Blum S. (2015) MatrixDB, the extracellular matrix interaction database: updated content, a new navigator and expanded functionalities. Nucleic Acids Res. 43, D321–D327 25378329 10.1093/nar/gku1091 PMC4383919

[52] Toukach P. V., and Egorova K. S. (2016) Carbohydrate structure database merged from bacterial, archaeal, plant and fungal parts. Nucleic Acids Res. 44, D1229–D1236 26286194 10.1093/nar/gkv840 PMC4702937

[53] Ceroni A., Dell A., and Haslam S. M. (2007) The GlycanBuilder: a fast, intuitive and flexible software tool for building and displaying glycan structures. Source Code Biol. Med. 2, 3 17683623 10.1186/1751-0473-2-3 PMC1994674

[54] Sehnal D., Deshpande M., Vařeková R. S., Mir S., Berka K., Midlik A., Pravda L., Velankar S., and Koîca J. (2017) LiteMol suite: interactive web-based visualization of large-scale macromolecular structure data. Nat. Methods 14, 1121–1122 29190272 10.1038/nmeth.4499

[55] Burley S. K., Berman H. M., Kleywegt G. J., Markley J. L., Nakamura H., and Velankar S. (2017) in Protein Crystallography, eds Wlodawer A, Dauter Z, Jaskolski M (Springer New York, New York, NY), pp 627–641

[56] Lee L. Y., Moh E. S. X., Parker B. L., Bern M., Packer N. H., and Thaysen-Andersen M. (2016) Toward automated *N* -glycopeptide identification in glycoproteomics. J. Proteome Res. 15, 3904–3915 27519006 10.1021/acs.jproteome.6b00438

[57] Liu G., Cheng K., Lo C. Y., Li J., Qu J., and Neelamegham S. (2017) A comprehensive, open-source platform for mass spectrometry-based glycoproteomics data analysis. Mol. Cell Proteomics 16, 2032–2047 28887379 10.1074/mcp.M117.068239 PMC5672007

[58] Chalkley R. J., and Baker P. R. (2017) Use of a glycosylation site database to improve glycopeptide identification from complex mixtures. Anal. Bioanal. Chem. 409, 571–577 27722944 10.1007/s00216-016-9981-2

[59] Wuhrer M., Stam J. C., van de Geijn F. E., Koeleman C. A. M., Verrips C. T., Dolhain R. J. E. M., Hokke C. H., and Deelder A. M. (2007) Glycosylation profiling of immunoglobulin G (IgG) subclasses from human serum. PROTEOMICS 7, 4070–4081 17994628 10.1002/pmic.200700289

[60] Horlacher O., Jin C., Alocci D., Mariethoz J., Müller M., Karlsson N. G., and Lisacek F. (2017) Glycoforest 1.0. Anal. Chem. 89, 10932–10940 28901741 10.1021/acs.analchem.7b02754

