# Supplementary material for: Glycomics@ExPASy: Bridging the Gap
Source: Mol Cell Proteomics. 2018 Aug 10;17(11):2164–76. doi: 10.1074/mcp.RA118.000799 (PMC6210229; doi:10.1074/mcp.RA118.000799)
Supplement: supplemental Table S1 [file RA118.000799_index.html]

Supplement to Glycomics@ExPASy: Bridging the gap | Molecular & Cellular Proteomics

## Supplemental Data

- supplemental figure - information on formats
- Supplementary tables - Tables containing further information on bioinformatics resources
