# Supplementary figures and images for: Glycomics@ExPASy: Bridging the Gap
Source: Mol Cell Proteomics. 2018 Aug 10;17(11):2164–76. doi: 10.1074/mcp.RA118.000799 (PMC6210229; doi:10.1074/mcp.RA118.000799)

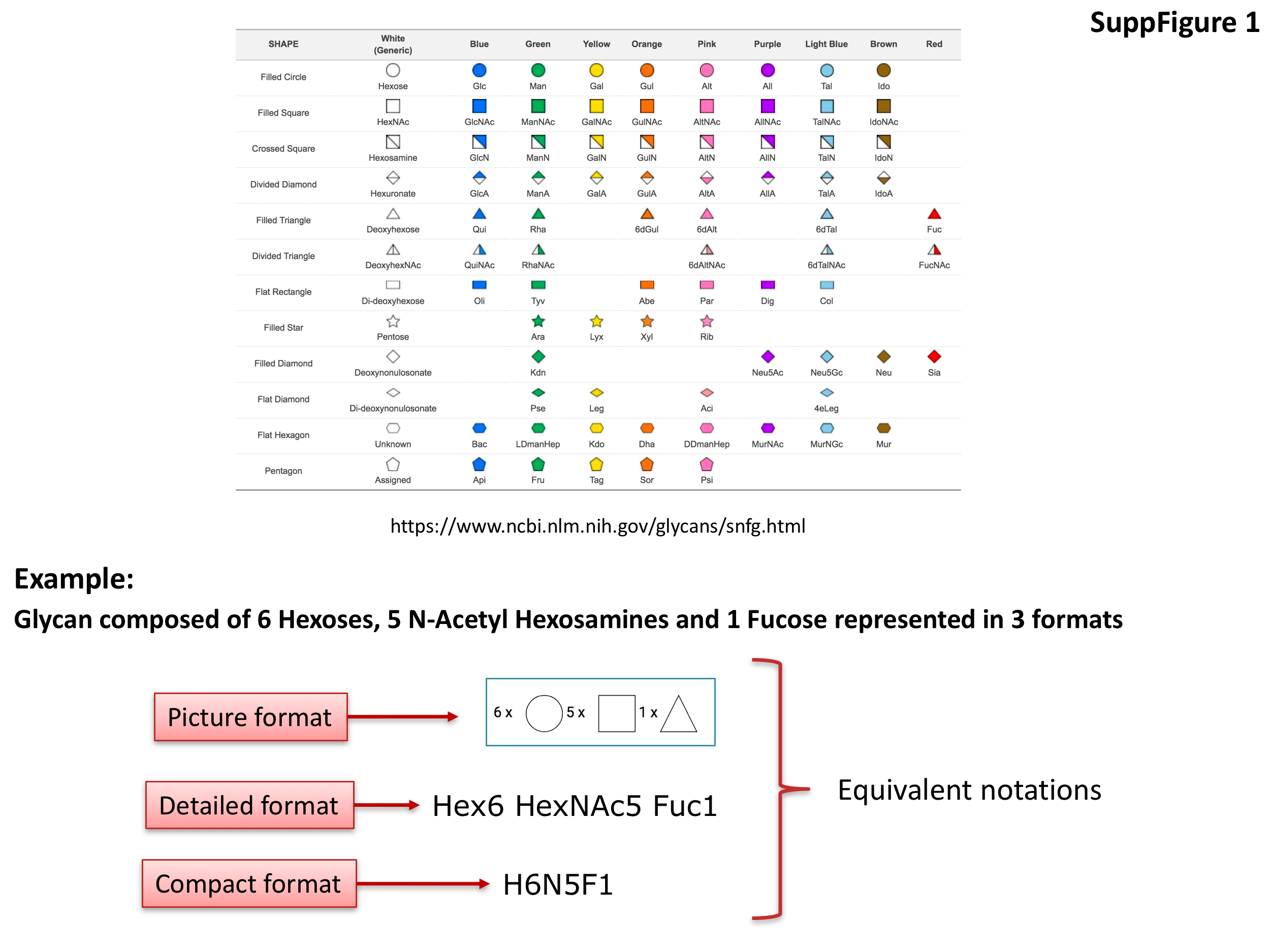

Supplement: supplemental Table S1 [file 137422_1_supp_169006_pd95j4.tif]
