# Supplementary material for: Glycomics@ExPASy: Bridging the Gap
Source: Mol Cell Proteomics. 2018 Aug 10;17(11):2164–76. doi: 10.1074/mcp.RA118.000799 (PMC6210229; doi:10.1074/mcp.RA118.000799)
Supplement: supplemental Table S1 [file 137422_1_supp_168460_pbtbf0.pdf]

Supplemental Material

Table S1. External resource table

This table lists all the external resources. For each, the table shows a short description followed by its reference.

Name	Purpose	Ref
**CAZy**	Database and classification of carbohydrate-active enzymes	(45)
**Unilectin**	Lectin information portal	-
**CSDB**	Carbohydrate Structure Database with a focus on Bacterial & Archeal (BCSDB) and Plant & Fungal (PFCSDB)	(52)
**GlyTouCan**	International glycan structure repository	(15)
**MatrixDB**	Database of interactions between extracellular matrix proteins, proteoglycans and polysaccharides	(51)
**Glyco3D**	Databases of 3D features of mono, oligo and polysaccharides, glycosyltransferases, lectins, mAbs against glycans, glycosaminoglycan-binding proteins	(38)
**DrawGlycan**	Conversion of IUPAC strings into SNFG cartoons	(61)
**GlycoDomain Viewer**	Visual browser for glycoproteomic data	(62)
**GLYCAM-web**	Toolbox for automating the prediction of 3D structures of glycans, glycosaminoglycans, and glycoproteins	-
**O-Glycologue**	Simulator of O-linked glycan synthesis	(63)
**NetCGlyc**	Neural network predictions of C-mannosylation sites in mammalian proteins	(64)
**NetOGlyc**	Neural network predictions of mucin type GalNAc O-glycosylation sites in mammalian proteins	(64)
**NetNGlyc**	Neural network predictions of N-Glycosylation sites in human proteins	(64)
**YinOYang**	Neural network predictions for O-beta-GlcNAc attachment sites in eukaryotic protein sequences.	(64)

Additional references:

61. Cheng, K., Zhou, Y., and Neelamegham, S. (2016) DrawGlycan-SNFG: a robust tool to render glycans and glycopeptides with fragmentation information. Glycobiology 27, 200–205

62. Joshi, H. J., J.rgensen, A., Schjoldager, K. T., Halim, A., Dworkin, L. A., Steentoft, C., Wandall, H. H., Clausen, H., and Vakhrushev, S. Y. (2018) GlycoDomainViewer: a bioinformatics tool for contextual exploration of glycoproteomes. Glycobiology 28, 131–136

63. McDonald, A. G., Tipton, K. F., and Davey, G. P. (2016) A Knowledge-Based System for Display and Prediction of O-Glycosylation Network Behaviour in Response to Enzyme Knockouts. PLOS Comput. Biol. 12, e1004844

64. Jensen, L. J., Gupta, R., Blom, N., Devos, D., Tamames, J., Kesmir, C., Nielsen, H., Staerfeldt, H. H., Rapacki, K., Workman, C., Andersen, C. A. F., Knudsen, S., Krogh, A., Valencia, A., and Brunak, S. (2002) Prediction of Human Protein Function from Post-translational Modifications and Localization Features. J. Mol. Biol. 319, 1257–1265

Table S2. Glycan properties table

This table lists the properties as originally defined in (25) and used in GlyConnect to search the database.

Category	Property type	Property name
Compositional	Fucosylation	Fucosylated
		Core-fucosylated
		Non-fucosylated
		Non-core-fucosylated
Compositional	Galactosylation	Galactosylated
		Non-galactosylated
Compositional	Sialylation	Sialylated
		Non-sialylated
		Mono-sialylated
		Di-sialylated
		Over-two-sialylated
Compositional	Xylosylation	Xylosylated
		Core-xylosylated
		Non-xylosylated
		Non-core-xylosylated
Structural	Antenna	Bi-antennary
		Tri-antennary
		Tetra-antennary
		Over-tetra-antennary
Structural	Bisection	Bisecting
		Non-bisecting
Structural	Truncation	Truncated
		Non-truncated
